# Possibility of designing catalysts beyond the traditional volcano curve: a theoretical framework for multi-phase surfaces†
†Electronic supplementary information (ESI) available. See DOI: 10.1039/c5sc01732g


**DOI:** 10.1039/c5sc01732g

**Published:** 2015-06-22

**Authors:** Ziyun Wang, Hai-Feng Wang, P. Hu

**Affiliations:** a Key Laboratory for Advanced Materials , Center for Computational Chemistry and Research Institute of Industrial Catalysis , East China University of Science and Technology , Shanghai 200237 , P. R. China; b School of Chemistry and Chemical Engineering , Queen's University Belfast , Belfast BT9 5AG , UK . Email: p.hu@qub.ac.uk

## Abstract

The current theory of catalyst activity in heterogeneous catalysis is mainly obtained from the study of catalysts with mono-phases, while most catalysts in real systems consist of multi-phases, the understanding of which is far short of chemists' expectation. Density functional theory (DFT) and micro-kinetics simulations are used to investigate the activities of six mono-phase and nine bi-phase catalysts, using CO hydrogenation that is arguably the most typical reaction in heterogeneous catalysis. Excellent activities that are beyond the activity peak of traditional mono-phase volcano curves are found on some bi-phase surfaces. By analyzing these results, a new framework to understand the unexpected activities of bi-phase surfaces is proposed. Based on the framework, several principles for the design of multi-phase catalysts are suggested. The theoretical framework extends the traditional catalysis theory to understand more complex systems.

## Abstract 2

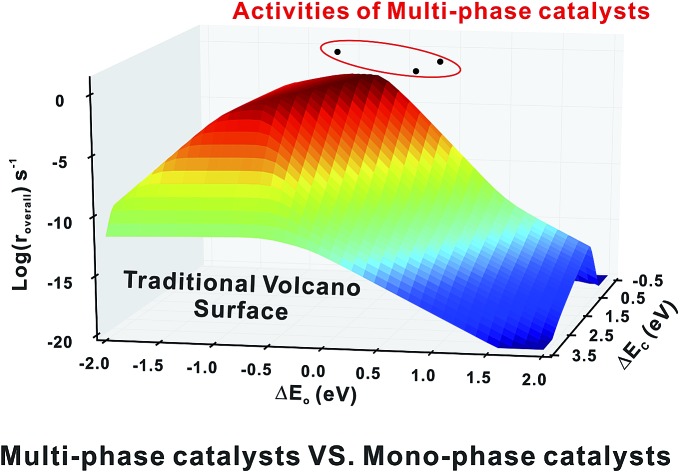
The current theory of catalyst activity in heterogeneous catalysis is mainly obtained from the study of catalysts with mono-phases, while most catalysts in real systems consist of multi-phases, the understanding of which is far short of chemists' expectation.

## Introduction

The volcano curve is one of the most important findings in heterogeneous catalysis: when the activities of catalysts are plotted across the periodic table, a curve with a volcano shape is usually obtained.^1^ Namely, the catalysts on the left hand side of the periodic table are usually not good and the catalysts on the right hand side are normally not good either; the catalysts in the middle are the best for the reaction. Recently, thanks to DFT calculations,^2^–^5^ the volcano curve has been explained on the molecular level with greater clarity: an inert catalyst is difficult to activate the reactants, resulting in high barriers in adsorption steps, while high desorption barriers are found for active catalysts.^4^,^5^ Hence, a good catalyst possesses a reasonable balance between the adsorption and desorption barriers. Currently, the volcano curve has been extensively used to guide new catalyst design.^3^,^6^–^8^ One may ask whether it is possible to break the constraints to develop better catalysts which possess low adsorption barriers as well as low desorption barriers. In this work, we show that adding more phases may be a promising approach to designing better catalysts beyond the traditional volcano curve of mono-phase catalysts.

In real catalytic systems, catalysts usually contain several phases. Some previous studies reported that certain multi-phase catalysts had outstanding activity.^9^–^14^ For example, Bao and co-workers published a series of papers^9^–^11^ on the bi-phasic surfaces for CO oxidation and several different bi-phase catalysts, such as Co–Pt,^9^ Ni–Pt^10^ and Fe–Pt,^11^ were investigated and excellent activities were found. Hu and co-workers^12^ reported that the bimetallic Pt–Re catalysts could catalyse hydrogenation of tertiary amides at low temperatures and pressures with outstanding activity. Using DFT calculations, they found that the high activity is due to the decrease of hydrogenation barriers by the two-phase catalyst. Furthermore, the cobalt molybdenum bimetallic nitride catalyst was reported to possess higher activity for ammonia synthesis than a doubly promoted iron catalyst.^13^,^15^,^16^ Based on this result, Nørskov and co-workers^3^ suggested an interpolation in the periodic table may be one promising approach to design bimetallic catalysts. In our previous work,^14^ it was proposed that due to the constraint between the adsorption energies of carbon and oxygen atoms on mono-phase catalysts, the catalysts with activity at the peak point of a three-dimensional volcano surface could only be reached by adding more phases. Based on the work mentioned above, it is clear that the multi-phase catalysts have some outstanding properties, which could enhance the catalytic activity for some reactions. However, there are still some general questions remaining to be answered: what unusual properties do the multi-phase catalysts possess resulting in the high activity? What kind of combinations could result in a good multi-phase catalyst? Is there a general framework to understand the multi-phase catalysts?

In order to address the questions mentioned above, a systematic investigation was carried out on nine different bi-phase surfaces using CO hydrogenation (CO + 3H_2_ → CH_4_ + H_2_O) as a model reaction. These systems were chosen for the following reasons: firstly, the bi-phase surfaces were used to represent multi-phase catalysts: this is because the bi-phase surfaces are the simplest multi-phase surfaces, and many multi-phase surfaces could be considered as combinations of bi-phase surfaces. Therefore, bi-phase surfaces are building blocks and may possess the general properties of multi-phase surfaces. Secondly, in this work, six different metals (Cu, Pd, Pt, Rh, Ru and Re), ranging from very noble to reactive metals, were combined in pairs to construct nine bi-phase surfaces. These bi-phase systems included all the possible combinations, such as noble–reactive, reactive–noble, noble–noble and reactive–reactive surfaces. Hence, a systematic study on these surfaces may give rise to a comprehensive account of multi-phase catalysts. Thirdly, CO hydrogenation into methane was chosen since this is a typical multistep surface reaction including the dissociation of strong CO bond and hydrogenation reactions. Therefore, some of the findings in this reaction may be general to heterogeneous catalysis.

In the current work, all the possible reactions were taken into account and calculated on all the nine bi-phase surfaces mentioned above using DFT. The activities of bi-phase surfaces were evaluated with micro-kinetic modelling.^17^,^18^ Compared to their corresponding mono-phase surfaces, extraordinary activities were found. The effects of different kinds of reactions on the activities were decomposed by using three different micro-kinetic models, and a new framework was developed and discussed to explain the activities.

## Computational details

**Fig. 1:**
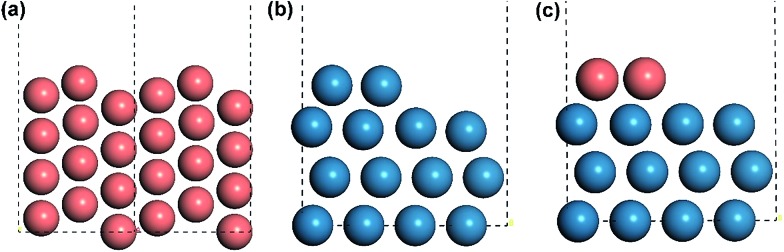
Illustration of the structures of stepped surfaces and interfaces. (a) Side view of the fcc mono-metal stepped Cu(211) surface, (b) the hcp mono-metal stepped Re surface and (c) the interface of CuRe bi-phase surface. Copper and rhenium atoms are in light red and light blue, respectively. The unit cells are marked with black dashed lines.

All the DFT calculations were carried out with a periodic slab model using the Vienna *ab initio* simulation program (VASP).^19^–^22^ The generalized gradient approximation (GGA) was used with Perdew–Burke–Ernzerhof (PBE)^23^ exchange–correlation functional. The projector-augmented wave (PAW) method^24^,^25^ was utilized to describe the electron–ion interactions and the cut-off energy of the plane-wave basis set was set to be 400 eV. 3 × 3 × 1 Monkhorst–Pack *k*-point mesh sampling was used for Brillouin zone integration. All the adsorption geometries were optimized using a force-based conjugate gradient algorithm, while transition states (TSs) were located with a constrained minimisation technique.^26^–^28^ For fcc mono-phase metal surfaces (Cu, Pd, Pt, Rh), (211) planes were chosen as the model catalysts with monoatomic steps. 12-layer models with *p*(1 × 3) supercell (as shown in Fig. 1(a)) were used for these surfaces with the lower 6 layer fixed and the 6 upper layers relaxed. For hcp mono-phase metal surfaces (Re and Ru), the monoatomic step was modelled by removing two neighbouring rows of metal atoms in the top layer on close-packed (001) surface, as used in our previous work^5^,^29^–^31^ (see Fig. 1(b)). For these surfaces, 4-layer *p*(3 × 4) supercell models were used with the 2 lower layers fixed and the 2 upper layers relaxed.

**Fig. 2:**
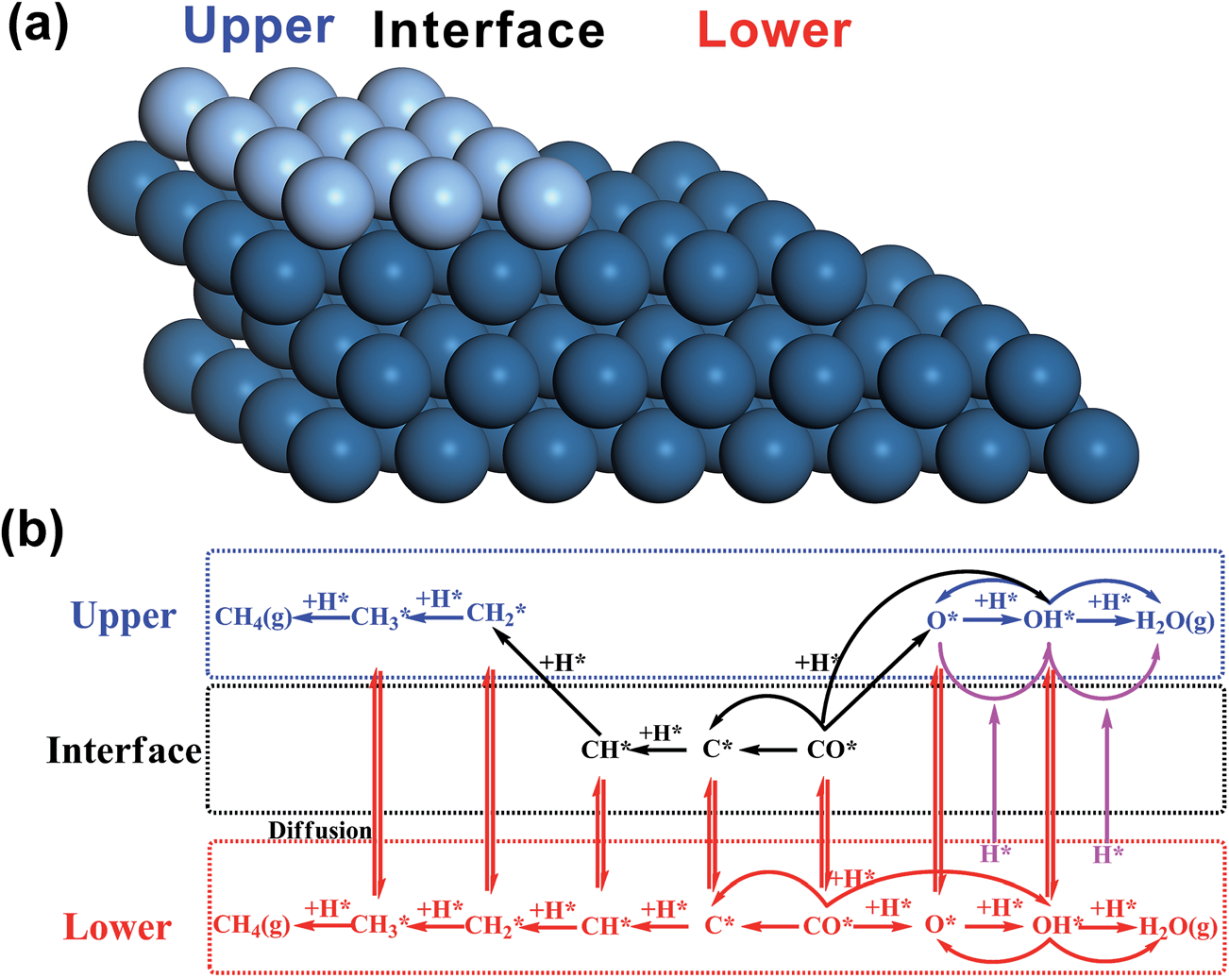
Scheme diagrams of a bi-phase surface AB (a) and the reaction network (b). In the surface model (a), the grey balls and blue balls are upper-phase metal atoms A and lower-phase metal atoms B, respectively. In the reaction network (b), the reactions on different phases are highlighted in different colours: blue on the upper phase, black on the interface and red on the lower phase. The interface hydrogenation reactions are shown in purple and defined in the main text. The diffusion is indicated.

Before introducing our computation models of bi-phase surfaces, it is worthwhile clarifying what bi-phase surfaces may be in real systems. In a bi-phase system with two types of metals A and B, the surfaces may either be mixed forming an alloy, or segregated forming mono-metal islands, where the percentages are determined by the surface segregation energies of A and B as suggested by Nørskov and co-workers.^32^,^33^ In other words, the bi-phase surfaces should include the mixed AB alloys, the segregated mono-metal islands A and B, and the interfaces between these islands. While the mixed alloys can be treated as mono-phases and have been widely studied,^8^,^34^–^42^ the outstanding activities of bi-phase surfaces may derive from the synergistic effect of different islands and the interfaces between them. Currently, the understanding of the activity of segregated mono-metal islands and the interfaces between islands is far short of general expectations. Therefore, a bi-phase surface model AB with three different phases was chosen, with upper terrace A, the interface between A and B, and lower terrace B with a monoatomic step, as shown in Fig. 2(a). For the modelling of bi-phase surface AB, we replaced the upper terrace atoms of the stepped B surface by A atoms. It is worth noting that some of the bi-phase surfaces may be not stable under real catalytic conditions; for example, for two types of metals, A and B, if A is stable on the B surface, B is likely not to be stable on A.^32^ However, to systematically and consistently investigate the activity of bi-phase surfaces, both AB and BA were considered and the optimizations of all the bi-phase surfaces were carried out using a force-based conjugate gradient algorithm.

In the model, the upper terrace A and the lower terrace B represent approximately the A and B metal phases, respectively, while the stepped interface between A and B denotes a junction between the A and B phases. This type of junction may have similar local structures as some alloys and hence it may possess some characteristics of AB alloys. More importantly, our recent theoretical work^12^ and also experimental work^9^–^11^ showed that this type of junction possesses unique activity. Therefore, our model may be general for investigation of bi-phase systems. In our calculations, the reactions on lower phase B were calculated on the pure stepped B surface, while the reactions on upper terrace A and the interface between A and B were calculated using the model in Fig. 1(c), which was constructed as follows: the close-packed flat surface B ((111) surface for fcc metal and (001) surface for hcp) was chosen with 4-layer *p*(3 × 4) supercell and then the two neighbouring rows of metal atoms in the top layer were removed, and the other two rows were replaced by metal A. The bottom two layers were fixed, while the upper two layers were relaxed. An ∼10 Å vacuum region was placed on all the models mentioned above. In this way, all the bi-phase surfaces were modelled using the same approach in this work to keep the consistency (the surface structures can be found in ESI†).

The mechanisms on mono-phase surfaces developed by Nørskov and co-workers^4^,^43^,^44^ were well-received, including the CO dissociation and the hydrogenation of carbon and oxygen containing species (reactions on the lower phase in Fig. 1(b)). Based on their mechanisms, for the bi-phase surfaces, the reactions on different phases and the diffusion of adsorbates were all considered: the reactions on the upper, interface and lower surfaces were all taken into account (Fig. 2(b)), and the diffusion between different phases were also included. In general, all the reactions can be divided into three groups: the mono-phase reactions, the interface hydrogenation reactions and the reactions on the lower phase. The mono-phase reactions refer to the reactions similar to those on the mono-phase surfaces^4^,^43^,^44^ (blue and black reactions in Fig. 1(b)). The interface hydrogenation reactions are the oxygen-containing species on the interface being hydrogenated by hydrogen on the lower phase^12^ (purple reactions in Fig. 1(b)). The lower phase reactions include all the mono-phase reactions on the lower mono-phase stepped surfaces. In this work, to keep all the kinetic results consistent, we treated the mono-phase surface as a special bi-phase surface, namely a bi-phase surface with the same metal for both upper and lower phase. Thus, the same kinetic model was used for both mono-phase and bi-phase surfaces. All the energies were converted into free energies using thermodynamic analysis^45^–^47^ and based on these free energies data micro-kinetic modelling was carried out using CatMAP code^48^ developed in Nørskov's group (see calculation details in the ESI†).

## Results and discussion

### Activities of bi-phase surfaces

**Fig. 3:**
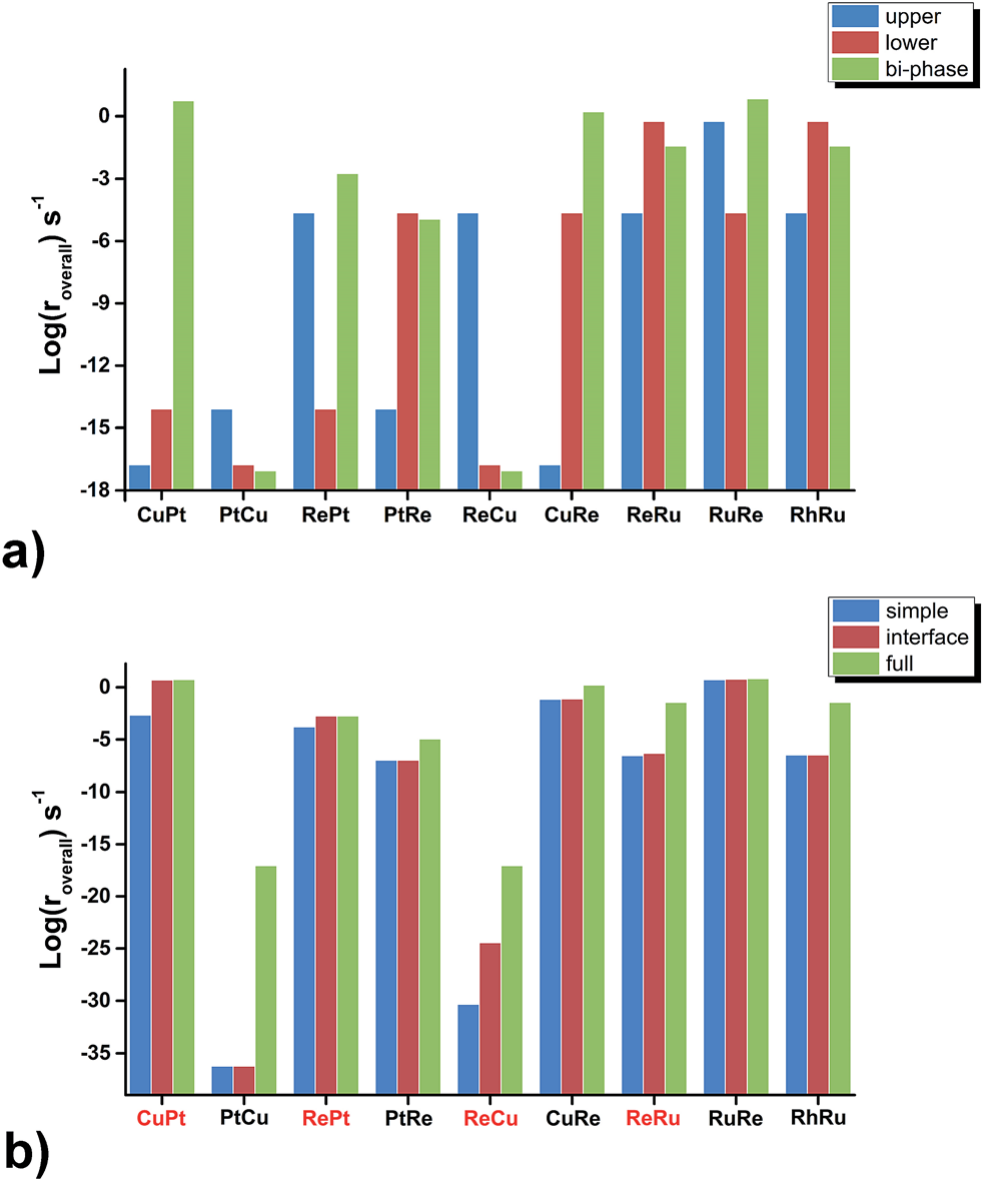
Activity (log(*r*_overall_)) comparisons of nine bi-phase surfaces among (a) those from the upper phase, lower phase and bi-phase surfaces, respectively, (b) the simple, interface and full kinetic models, where the reactive–noble bi-phase surfaces are highlighted in red.

**Fig. 4:**
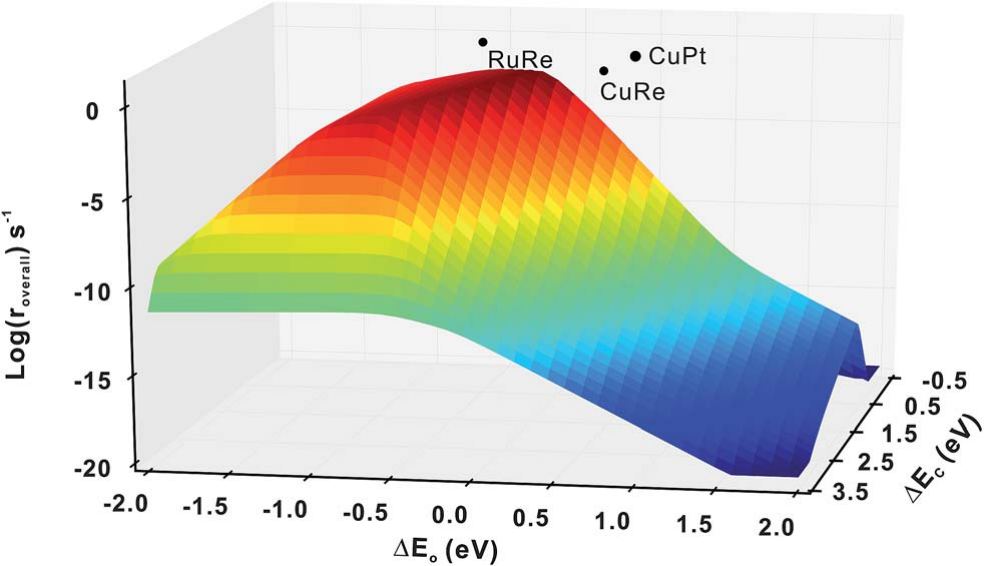
Comparison of activities (log(*r*_overall_)) between the mono-phase volcano surface and the three bi-phase surfaces, RuRe, CuRe and CuPt, illustrating that some bi-phase systems can achieve activities far beyond the mono-phase volcano surface.

Firstly, we compare the activities of bi-phase surfaces with those of their corresponding mono-phase surfaces, as shown in Fig. 3(a), and the overall reaction rates of the nine different bi-phase surfaces are listed in Table 1 (column full). From these activity values, several striking features can be found: firstly, the compositions of the reactive bi-phase surfaces are very different: stacking Ru (an reactive mono-phase surface; the metals on the left hand side of the periodic table are defined as the reactive ones and the metals on the right hand side are defined as the noble ones in this work) on Re (a reactive metal) results in the most reactive bi-phase catalyst (RuRe), while CuRe and CuPt which are also very reactive are assembled by intrinsic noble–reactive and noble–noble stackings, respectively. These high activities of noble–reactive and particularly intrinsic noble–noble composition combinations seem to be unexpected. Secondly, the stacking order of the two phases can influence the activity dramatically. For example, with the same compositions but different stacking orders, the activity of RuRe is more than two orders of magnitude higher than that of ReRu (Table 1). Thirdly, in some cases, the activities are significantly enhanced when two phases are combined, such as CuPt and CuRe (Fig. 3(a)). However, in other cases, the activities of bi-phases are similar to those of their mono-phase surfaces. Perhaps more importantly, the activities of several bi-phase systems are very high, compared with their corresponding mono-phase surfaces. For example, Fig. 4 shows clearly that the activities of CuPt, CuRe and RuRe are several orders of magnitude beyond the traditional volcano surface. These unexpected results are difficult to understand using the existing catalysis theories. A new theoretical framework is needed to explain the trends of these activities and to predict the compositions and stacking orders of bi-phase surfaces for new catalyst design.

**Table 1:** Reaction rates of CO hydrogenation on nine bi-phase surfaces using three different micro-kinetic models (simple for kinetic model without interface hydrogenation, interface for the kinetic model with the interface hydrogenation included but without diffusion and full for the kinetic model including all the reactions and diffusion shown in Fig. 2(b)). All the reaction rates are in s^–1^

	Simple	Interface	Full
CuPt	2.17 × 10^–3^	4.97	5.53
PtCu	6.11 × 10^–37^	6.11 × 10^–37^	8.80 × 10^–18^
RePt	1.62 × 10^–4^	1.82 × 10^–3^	1.82 × 10^–3^
PtRe	1.17 × 10^–7^	1.17 × 10^–7^	1.14 × 10^–5^
ReCu	4.55 × 10^–31^	3.59 × 10^–25^	8.80 × 10^–18^
CuRe	7.23 × 10^–2^	7.77 × 10^–2^	1.63
ReRu	3.24 × 10^–7^	5.29 × 10^–7^	3.63 × 10^–2^
RuRe	5.48	6.09	6.90
RhRu	3.65 × 10^–7^	3.65 × 10^–7^	3.64 × 10^–2^

### Kinetic characteristics

In order to understand the results above, we compared the differences between the bi-phase and mono-phase surfaces, and the following aspects were found to be related to the outstanding activities:

(i) The structures of bi-phase surfaces may change the chemisorption energies, and thus the high activities of some bi-phase surfaces may derive from the appropriate adsorption properties. It is called the structural effect in this work. This effect can be illustrated using a simple kinetic model similar to the simple one for mono-phase surfaces, *i.e.* the kinetic model without interface hydrogenation, lower phase reaction and diffusion.

(ii) On some bi-phase surfaces, the hydrogenation reactions are slow while the interface sites can speed up the hydrogenation considerably, resulting in the high activities. This is called the energetic effect, and can be decomposed by comparing the activity differences between the simple kinetic model in section (i) with and without the interface hydrogenation.

(iii) In bi-phase systems, in addition to the reaction sites on the bi-phase interface, the reactions can also occur on lower metal phases. Furthermore, the diffusion between the two phases may also enhance the activity. These two effects, namely the existence of lower phase and the diffusion effect, may affect the activities of bi-phase surfaces. These two effects often come together and can be evaluated by comparing the activities from the interface kinetic model in section (ii) and the full kinetic model with all reactions. The combination of these two effects is called the kinetic effect.

### Structural effect

**Fig. 5:**
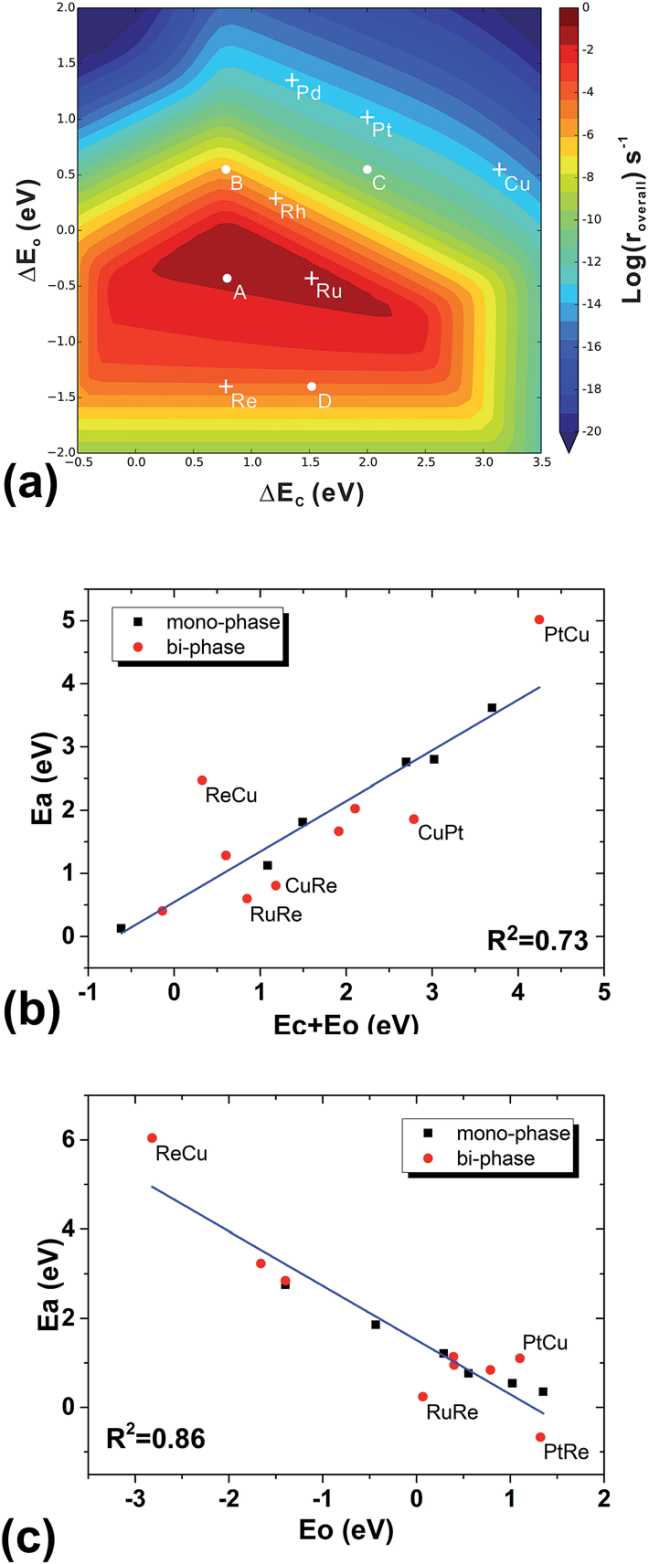
The volcano surface of activity from the simple kinetic model without diffusion and interface hydrogenation for mono-phase catalysts (a), the BEP relation between the barriers of CO dissociation and the adsorption energies of carbon and oxygen (b), and the BEP relation between the effective barriers of oxygen desorption steps and the adsorption energies of oxygen (c). DFT data from both mono-phases (black) and bi-phases (red) are used in (a) and (b). For the mono-phase surfaces, the adsorption energies correspond to those of carbon on B5 sites and oxygen on the upper terrace. For the bi-phase surfaces, the adsorption energies of carbon on the B5 sites of pure lower-phases and the adsorption energies of oxygen on the upper terraces of bi-phase surfaces are used. The points that fall far from the line are labelled.

To understand the structural effect, we need first to simplify our reaction kinetics to pin down the key factors in the seemingly complicated systems. We find the following evidence that shows that our bi-phase systems can be qualitatively described by the kinetic framework of traditional mono-phase surfaces. Firstly, we find that the overall reaction can be decomposed into three parts: (i) the C–O bond dissociation which is either the direct C–O breaking or the H-assisted C–O scissoring; (ii) the CH_4_ formation including the hydrogenation of C_1_ (CH_*x*_) and CH_4_ desorption; and (iii) the H_2_O formation including the hydrogenation of O-containing species and H_2_O desorption. Furthermore, we find that the rate-determining steps are either the C–O activation or the H_2_O formation. In other words, the total rates are determined by the competition between the C–O dissociation and the H_2_O formation; a low effective C–O dissociation barrier leads to a high effective barrier of H_2_O formation or *vice versa*. This is consistent with the kinetic study on mono-phase catalysts.^14^ Secondly, in the mono-phase kinetic model, the overall reaction rate is determined by two BEP relations; the C–O dissociation barrier *vs.* the sum of the adsorption energies of carbon and oxygen atoms, and the H_2_O formation barrier *vs.* the adsorption energy of oxygen atoms, from which the 3-D volcano surfaces can be obtained, as shown in Fig. 5(a). By examining the TSs of C–O dissociation, we find that on all the bi-phase catalysts, the C–O activation possesses similar geometries: O is always on the bridge site of the step edge of the interface while C is on the lower phase. When the effective C–O dissociation barriers from the bi-phase systems are plotted against the sums of the carbon adsorption energies on the lower mono-phase surfaces and the oxygen adsorption energies on the bridge sites of the step edge of bi-phase surfaces, a linear relation is obtained (Fig. 5(b)), which includes DFT data from mono-phases. Similarly, the effective barriers of H_2_O formation from the bi-phase systems are also found to correlate linearly with the adsorption energies of oxygen atoms on the corresponding bi-phases (Fig. 5(c)). This suggests that the main kinetic features of the bi-phase systems are similar to those from the mono-phase kinetics, *i.e.* the overall rates of bi-phase surfaces are also related to the adsorption energies on their corresponding sites.

### Understanding of the stacking orders

To further check the results above, we ran a simplified micro-kinetic calculation for our bi-phase systems with the same reaction network as that on mono-phases (black and blue reactions in Fig. 2(b)). The interface hydrogenation and diffusion were ignored (their effects will be analysed later). The reactions on the lower phase were not considered. This is in fact the kinetic model that was well tested for CO hydrogenation by Nørskov and co-workers for mono-phase surfaces.^4^,^43^,^44^ The reaction rates of our bi-phase systems from this simple kinetic model are listed in Table 1. It can indeed be seen that the activities from the simple kinetic model and those of the full kinetics model have similar trends. Therefore, the simplified kinetics model should allow us to identify the key features in our systems.

Based on the discussion in the section of structural effect, the reaction rates of bi-phase surfaces from the simple kinetic model without the interface hydrogenation and diffusion are related to the adsorption energies of O on the step edge of bi-phase surfaces and C of the lower mono-phase surface. As shown in Fig. S1,† the adsorption energy of O on the step edge of a bi-phase surface, namely AB, is linearly correlated with the adsorption energy of O on the same site of the mono-phase A. Therefore, we can provide a simple explanation for the reaction rates of bi-phase surfaces with the adsorption energies of mono-phase only, using which the compositions and the stacking orders in the bi-phase systems can be explained: taking RuRe as an example, the approximate position of activity of RuRe is point A in Fig. 5(a), with the adsorption energy of carbon atom on the lower phase (Re) and the adsorption energy of oxygen atom on the upper terrace (Ru). Therefore, RuRe is closer to the peak of the volcano surface (Fig. 5(a)) and thus more active than Ru. However, the rate for the inversed surface, *i.e.* ReRu, would be around point B (Fig. 5(a)), with the adsorption energy of the carbon atom on the lower phase (Ru) and the adsorption energy of the oxygen atom on the upper terrace (Re), resulting in the lower activity. Similarly, point C in Fig. 5(a) should be the approximate reaction rate for another outstanding bi-phase surface, CuRe, which has a much better activity than their corresponding mono-phases alone.

### Energetic contributions to the activity

Although the trends in the bi-phase surfaces can be explained using simple kinetics, quantitatively the differences between the full kinetic model and the simple one are still substantial (Table 1). For example, the activity of CuPt from the full kinetic model is three orders of magnitudes higher than that from the simple kinetic model. In other words, the activities of some bi-phase systems are beyond the volcano surfaces of mono-phase catalysts. How can we understand this? To answer this question is very desirable and fundamental for new catalyst design. The question can be addressed by considering the differences between the full kinetic model and the simple one: there are two aspects in the full kinetic model, which are omitted in the simple kinetic model; the first one is the interface hydrogenation in the bi-phase systems and the other one is the existence of a lower phase surface and the diffusion of reaction intermediates between the upper and lower phases, which will be discussed point by point below.

Regarding the hydrogenation on the interface in the bi-phase systems, in the simple kinetic model we added the hydrogenation steps of O-containing species at the interfaces, named as the interface kinetic model in the current work (Fig. 2(b)). The reaction rates of the bi-phase systems from the interface kinetic model are listed in Table 1 (column interface) and are compared to those from the simple kinetic model (Fig. 3(b)). It can be seen from the figure that the rates of some surfaces (highlighted in red) increase dramatically due to interface hydrogenation (red bar) compared to those of the simple ones (blue bar), while others stay almost the same. It is also worth pointing out that only the surfaces with a reactive-noble stacking benefit from the new interface reactions.

**Fig. 6:**
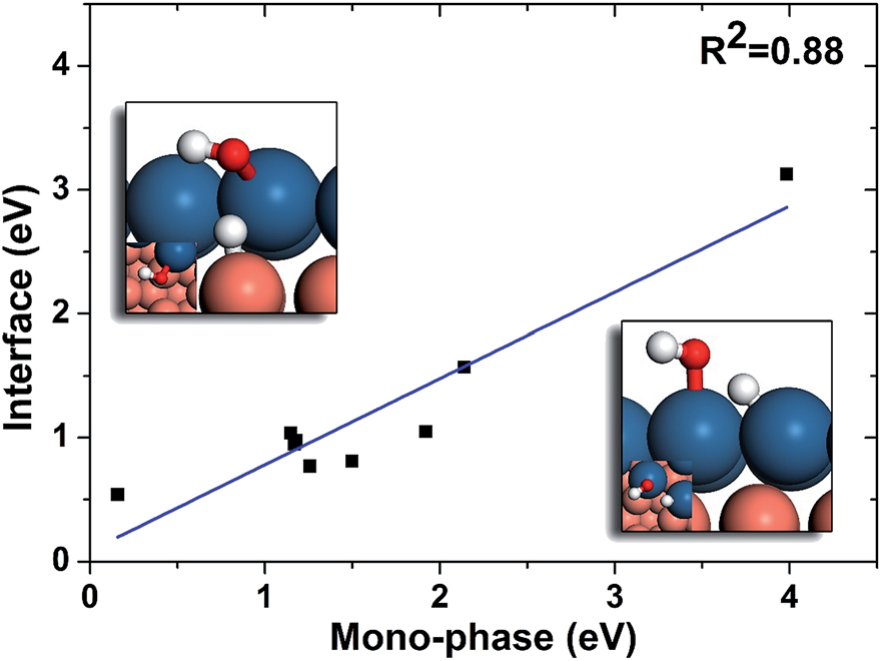
Linear relation between the barriers of OH hydrogenation on mono-phases and those of interface hydrogenation. The geometries of mono-phase hydrogenation of OH and OH hydrogenation on PtCu interface are shown on lower right and upper left, respectively. The blue, brown, white and red balls stand for Pt, Cu, H and O, respectively.

In order to further understand these results, we compared the reaction barriers of hydrogenation reactions at the interfaces to those on mono-phases, and the results are listed in Table 2. It can be seen that the hydrogenation reactions at the interfaces are much more favourable than those on the mono-phases. In fact, a correlation between the barriers of mono-phase hydrogenations of OH and those at the interfaces is found (Fig. 6). It can be seen that the interface reactions possess generally lower barriers than those on the corresponding mono-phases. Then, we analysed the effects of each elementary step on the overall reaction rate in the simple kinetic model using the degree of rate control (DRC) methods,^49^–^52^ which is an excellent tool in surface kinetics proposed by Campbell and co-workers to evaluate the effect of one reaction on the overall reaction rate. From the DRC results listed in Table S1,† the hydrogenation reactions of O-containing groups are found to have the largest DRC values, and thus are the rate-determining steps on all the surfaces with the intrinsic reactive-noble stacking order, while the other surfaces are limited by CO dissociation. This is because in the intrinsic reactive–noble systems, the high adsorption energies of oxygen on the upper phases after the CO dissociation result in high barriers of oxygen hydrogenation. As discussed above, the interfaces can enhance the hydrogenation of O-containing species (Fig. 6), and hence the intrinsic reactive-noble surfaces become more favourable with the interface hydrogenation steps considered in the interface kinetic model.

**Table 2:** Comparison of the reaction barriers of hydrogenation of O-containing species: the mono-phase hydrogenation and the interface hydrogenation on nine bi-phase surfaces. The unit is eV

	O* + H* → OH* + *		OH* + H* → H_2_O(g) + *	
	Mono	Interface	Mono	Interface
CuPt	1.16	0.83	1.50	0.81
CuRe	1.21	0.88	1.26	0.77
PtCu	1.26	0.15	1.17	0.95
PtRe	0.81	0.50	0.16	0.54
ReCu	4.47	2.80	3.98	3.13
RePt	1.71	0.56	1.92	1.05
ReRu	1.92	0.96	2.14	1.57
RhRu	1.18	0.44	1.18	0.98
RuRe	1.07	1.33	1.15	1.04

It is worth mentioning that recently, Vojvodic and Nørskov^53^ proposed a new paradigm for catalyst design by circumventing the scaling relations, in which four different effects for achieving this circumventing were proposed, namely the intrinsic, electronic, extrinsic, and structural effects. Interestingly, the interface structure was listed in the extrinsic effect. The current work shows that the interface can greatly decrease the activation energies of some reactions, and thus offers an example for such a design paradigm.

### Kinetic effects of two phases

Regarding intermediate diffusion on the surfaces, it is known that at typical reaction temperatures, surface diffusion can readily occur. On the mono-phase catalysts, the surface diffusion does not normally affect the overall reaction rates considerably. However, on the bi-phase surfaces as shown in Fig. 2(a), some reactions can occur on the upper phases and some on the lower phases through diffusion between the upper and lower phases. These reactions on both phases and diffusion may lead to some different activities on the bi-phase systems from the mono-phases.

To study the effects of the existence of the lower phase and diffusion on the activities, we compared the overall rates from the interface kinetic model to those of the full kinetic model in which the reactions on the lower phase and surface diffusion are allowed (Fig. 3(b)). We can see that the overall rates on all the intrinsic noble–reactive surfaces increase significantly with the full kinetic model compared to those from the interface kinetic model, while the reaction rates on the intrinsic reactive–noble surfaces are only slightly enhanced. This can be understood as follows: the rate-determining steps on the intrinsic noble–reactive surfaces are the CO dissociation reactions and the lower reactive phases in the intrinsic noble–reactive systems offer active sites for the CO dissociation, leading to activity enhancement of the intrinsic noble–reactive surfaces. However, due to the strong binding energies of carbon and oxygen atoms on the lower intrinsic reactive phases, the hydrogenation and desorption of the dissociated species are unfavourable. Therefore, the adsorbates would diffuse to more favourable sites for hydrogenation and desorption.

To further decompose the effects of simultaneous reactions and diffusion effects, we used a decoupled bi-phase approach: for a bi-phase surface AB, instead of calculating the activation energies and enthalpy changes for all the reactions explicitly on the bi-phase surface, we chose the related energies of pure A and pure B for the upper and lower phases, respectively, to carry out the micro-kinetic modelling. Three different levels of micro-kinetic models were used, namely the kinetic model with reactions on upper phases only, reactions on both phases without diffusion, and reactions on both phases with diffusion. Using this decoupled bi-phase approach with three levels of micro-kinetic models, the structural and energetic effects of the bi-phase surface can be excluded, and the effects of simultaneous reactions due to the existence of the lower mono-phase and diffusion effects can be de-convoluted. Namely, the effects of the existence of the lower phase can be obtained by comparing the reaction rates from the kinetic results with upper reactions only and those without diffusion, while the activity difference between the data with and without diffusion can represent the effects of diffusion. Similarly to the decoupled bi-phase approach, we also calculated the overall reaction rates from three different levels of micro-kinetic models using the actual energies from the interface bi-phases (named as the interface bi-phase in Table 3) in order to compare the results (named as the decoupled bi-phase in Table 3) from the decoupled bi-phase approach in which for a AB bi-phase the energies from pure A and pure B were used. From the table, several interesting features can be found: firstly, in general the activities from the decoupled approach and the interface approach have similar trends. However, the activities from the decoupled bi-phase approach are much lower than those from the interfacial bi-phase approach in general, suggesting that the structural and energetic effects of bi-phase surface mentioned above greatly enhance the reaction rates. Secondly, compared the reaction rates from the kinetic model with reactions on the upper phase and the model without diffusion (Table 3), for most bi-phase surfaces, the increases of activities due to kinetic effects can be attributed to the simultaneous reactions. For example, for ReRu, in the presence of lower Ru phase that is a reactive phase for CO hydrogenation, the activity is increased by 3 orders of magnitude. Thirdly, the diffusion between upper and lower phases also enhances the activity of some bi-phase surfaces. For example, the activities of CuRe from kinetic models without and with diffusion are 1.09 × 10^–4^ and 1.13 × 10^–5^, respectively, suggesting that the diffusion of intermediates can improve the activity of some bi-phase surfaces. Therefore, in the presence of diffusion and reaction on both phases, the two different phases provide an increased choice of reaction site for different types of reactions, resulting in higher activity.

**Table 3:** Reaction rates of CO hydrogenation from three different kinetic models, namely the kinetic models considering the reactions on upper phases only (A phase), the reactions on both A and B phases without diffusion, and the reactions on both A and B phases with diffusion, using the energies from pure A and pure B (column decoupled bi-phase approach) and the energies from the actual bi-phase AB (column interface bi-phase approach). All the reaction rates are in s^–1^

	Decoupled bi-phase approach			Interface bi-phase approach		
	Upper only	Without diffusion	With diffusion	Upper only	Without diffusion	With diffusion
CuPt	8.80 × 10^–18^	7.73 × 10^–16^	7.73 × 10^–16^	4.97	4.97	5.53
PtCu	7.64 × 10^–16^	7.73 × 10^–16^	4.13 × 10^–15^	6.11 × 10^–37^	8.80 × 10^–18^	8.80 × 10^–18^
RePt	1.13 × 10^–5^	1.13 × 10^–5^	1.13 × 10^–5^	1.82 × 10^–3^	1.82 × 10^–3^	1.82 × 10^–3^
PtRe	7.64 × 10^–16^	1.13 × 10^–5^	1.13 × 10^–5^	1.17 × 10^–7^	1.14 × 10^–5^	1.14 × 10^–5^
ReCu	1.13 × 10^–5^	1.13 × 10^–5^	1.09 × 10^–4^	3.59 × 10^–25^	8.80 × 10^–18^	8.80 × 10^–18^
CuRe	8.80 × 10^–18^	1.13 × 10^–5^	1.09 × 10^–4^	7.77 × 10^–2^	7.77 × 10^–2^	1.63
ReRu	1.13 × 10^–5^	3.63 × 10^–2^	3.63 × 10^–2^	5.29 × 10^–7^	3.63 × 10^–2^	3.63 × 10^–2^
RuRe	3.63 × 10^–2^	3.63 × 10^–2^	3.63 × 10^–2^	6.09	6.09	6.9
RhRu	2.21 × 10^–8^	3.63 × 10^–2^	3.63 × 10^–2^	3.65 × 10^–7^	3.63 × 10^–2^	3.64 × 10^–2^

### Implications for new catalyst design

Last but not least, there are some significant implications from our work for new catalyst design. What we have learned from the current work can be expressed as the following procedure for new catalyst design. (i) One may start with the traditional volcano surface from mono-phases, labelling all the mono-phase catalysts that may be used on the activity map (*i.e.* the TOF plots). (ii) To choose a good composition of bi-phases, we may consider two different phases with suitable carbon and oxygen adsorption energies according to the peak point of traditional mono-phase volcano surface, respectively, as shown in Fig. 5(a). (iii) To select the stacking order, one may choose the phase with the most suitable adsorption energies of oxygen to be the upper phase, and the one with the most suitable adsorption of carbon to be the lower phase. In such a way, new catalysts may be obtained with much higher activity compared to the traditional mono-phase system. This design procedure may be generally used in any type of reaction that contains two key intermediates. Furthermore, for reactions containing more than two key intermediates, one may consider multi-phase surfaces, and treat them as combinations of our bi-phase models.

## Conclusions

This work represents an attempt to systematically understand the extraordinary activity of bi-phase surfaces and explore the relation between the activity and the bi-phase structures (*i.e.* compositions and stacking orders). On nine bi-phase and six mono-phase surfaces, the elementary steps of CO hydrogenation were investigated using DFT calculations, and the activities were evaluated using micro-kinetics simulations based on DFT free energies. By comparing the activities of these bi-phase surfaces and their corresponding mono-phase surfaces, the following conclusions were obtained:

(i) The compositions of the active bi-phase surfaces are quite different.

(ii) The stacking order of bi-phase surfaces affects the activity dramatically.

(iii) The activities of some bi-phase surfaces are extraordinarily high and are beyond the traditional volcano surface.

In order to understand the unexpected activities of bi-phase surfaces, we used three levels of micro-kinetic models to decompose the effects of different contributions. The outstanding activities are found to derive from the following aspects:

(i) The combination of different mono-phase surfaces makes it possible to reach some points closer to the peak of the volcano surface.

(ii) The interface between two phases can lower the barriers of O-containing hydrogenation.

(iii) The simultaneous reactions on both phases and diffusion can provide a greater choice of reaction site for different reactions, which can speed up the overall reaction rate by several orders of magnitude in some cases.

Regarding the bi-phase catalyst design, some implications are obtained and a simple procedure for new catalyst development is proposed:

(i) For a given reaction, one needs to choose the most important two intermediates, for example C and O in this work, and plot the traditional mono-phase volcano surface related to the adsorption energies of these intermediates.

(ii) For the composition of bi-phase surfaces, two different phases may be considered according to the adsorption energies of the two key intermediates near the peak of the traditional volcano surface.

(iii) The stacking order of the two phases can be chosen according to the adsorption site preferences of the key intermediates, as mentioned in Section 3.6.

## Supplementary Material

Supplementary information

Supplementary information
