# Supplementary material for: Possibility of designing catalysts beyond the traditional volcano curve: a theoretical framework for multi-phase surfaces
Source: Chem Sci. 2015 Jun 22;6(10):5703–11. doi: 10.1039/c5sc01732g (PMC5947508; doi:10.1039/c5sc01732g)
Supplement: Supplementary file 2 [file SC-006-C5SC01732G-s002.pdf]

Electronic Supplementary Material (ESI) for Chemical Science.
This journal is © The Royal Society of Chemistry 2015

Supporting Information

Possibility of Designing Catalysts beyond the Traditional Volcano Curve: A Theoretical Framework for Multi-Phase Surfaces

Ziyun Wang,[a, b] Hai-Feng Wang[b] and P. Hu[a, b *]

[a]*Key Laboratory for Advanced Materials, Center for Computational Chemistry and Research Institute of Industrial Catalysis, East China University of Science and Technology, Shanghai 200237, P.R. China*

[b]*School of Chemistry and Chemical Engineering, Queen's University Belfast, Belfast BT9 5AG, U.K.*

1. Calculation details

1.1. Thermodynamic Analysis

Herein, some standard formulas of statistical mechanics were used to calculate the free energy corrections including zero-point-energy (ZPE), thermal energy and entropy derived from partition functions[1, 2].

For surface adsorbates, only vibrational contribution was considered, including ZPE, vibrational thermal energy and vibrational entropy. The ZPE correction is given by:

$$E_{ZPE} = \sum_i \frac{h\nu_i}{2} \tag{1}$$

where h is Planck's constant and v_i is vibrational frequency i which is calculated based on the harmonic oscillators approximation[3]. The standard molar vibrational thermal energy contribution is calculated by:

$$U^{\circ}_{vib} = RT \sum_i \frac{{h\nu_i}/{k_B}}{e^{{h\nu_i}/{k_B T}} - 1} \tag{2}$$

where R is the gas constant and k_B is Boltzmann's constant. The standard molar vibrational entropy is calculated using the following expression:

$$S^{\circ}_{vib} = R \sum_i [\frac{h\nu_i/k_B T}{e^{h\nu_i/k_B T} - 1} - \ln(1 - e^{h\nu_i/k_B T})] \tag{3}$$

Therefore, the standard molar Gibbs free energy for surface species in CO hydrogenation is obtained by:

$$G^{\circ} = E_{total} + E_{ZPE} + U^{\circ} - TS^{\circ} \tag{4}$$

where E_{total} refers to the total energy obtained from DFT calculation, and P is the partial pressure of the gas phase molecule. While thermodynamic corrections for the gaseous species were calculated from Shomate equation. These correlations were all obtained

using CatMAP[4-7] code. According to Nørskov's work[8], the reaction temperature was set to 523 K, and 0.01 bar, 0.97 bar, 0.01 bar and 0.01 bar were used for the partial pressures of CO, H_2, H_2O and CH_4, respectively.

1.2. Microkinetic Model

In this work, four reaction sites were assumed to involve in the reaction on mono-phase surface, namely the top of the step, the B5 site, the upper terrace and a hydrogen adsorption site[8]. The species on the top of the step included the CO*, OH* and O*, while low coordinated species (CH* and C*) were assumed to be adsorbed on the B5 site. The adsorption and hydrogenation of CH_2 and CH_3 were calculated on the upper terrace, and the hydrogen adsorption site was only available for hydrogen adsorption and dissociation.

As shown in Fig. 2 in manuscript, the microkinetic model of bi-phase surface AB includes three reaction sites: the upper terrace of A, the interface of AB and the lower stepped B. On the interface between A and B, the low coordinated species (CH* and C*) are adsorbed and hydrogenated similar to the B5 site mentioned in mono-phase surface. Furthermore, the CO dissociation was also calculated on the interface, due to the high activity of B5 site[9, 10]. Therefore, the following reactions were considered on interface ($*_u$ and $*_i$ stands for the reaction site on upper and interface, respectively; * is the hydrogen adsorption state mentioned above):

$CO*_u + *_i \leftrightarrow C*_i + O*_u$

$CO*_u + H* + *_i \leftrightarrow C*_i + OH*_u + *$

$C*_i + H* \leftrightarrow CH*_i + *$

$CH*_i + H* + *_u \leftrightarrow CH_2*_u + *_i + *$

The upper phase includes the top of step (O^* and OH^*) and the upper terrace (CH_2^* and CH_3^*), where the following reactions occur:

$H_2(g) + 2^* \leftrightarrow 2H^*$

$CO(g) + *_u \leftrightarrow CO*_u$

$CH_2*_u + H^* \leftrightarrow CH_3*_u + *$

$CH_3*_u + H^* \leftrightarrow CH_4(g) + * + *_u$

$O*_u + H^* \leftrightarrow OH*_u + *$

$OH*_u + H^* \leftrightarrow H_2O(g) + * + *_u$

$2OH*_u \leftrightarrow H_2O(g) + O*_u + *_u$

The reaction network on lower phase in the bi-phase system is the same as that of stepped mono-phase surface ($*_l$ stands for the site on lower phase and * is the hydrogen adsorption site on lower phase):

$CO(g) + *_l \leftrightarrow CO*_l$

$H_2(g) + 2^* \leftrightarrow 2H^*$

$CO*_l + *_l \leftrightarrow C*_l + O*_l$

$CO*_l + H^* + *_l \leftrightarrow C*_l + OH*_l$

$C*_l + H^* \leftrightarrow CH*_l + *$

$CH*_l + H^* \leftrightarrow CH_2*_l + *$

$CH_2*_l + H^* \leftrightarrow CH_3*_l + *$

$CH_3*_l + H^* \leftrightarrow CH_4(g) + * + *_l$

$O*_l + H^* \leftrightarrow OH*_l + *$

$OH*_l + H^* \leftrightarrow H_2O(g) + * + *_l$

$2OH*_l \leftrightarrow H_2O(g) + O*_l + *_l$

At last, the diffusion between these different phases were also taken into account:

$CH_3*_u + *_l \leftrightarrow CH_3*_l + *_u$

$CH_2*_u + *_l \leftrightarrow CH_2*_l + *_u$

$CH*_i + *_l \leftrightarrow CH*_l + *_i$

$C*_i + *_l \leftrightarrow C*_l + *_i$

$CO*_i + *_l \leftrightarrow CO*_l + *_i$

$O*_u + *_l \leftrightarrow O*_l + *_u$

$OH*_u + *_l \leftrightarrow OH*_l + *_u$

$H*_u + *_l \leftrightarrow H*_l + *_u$ (this is the hydrogen diffusion between the upper and lower hydrogen adsorption site)

The microkinetic models of both mono-phase and bi-phase surfaces were solved using self-consistent mean field approach. Steady-state solutions of microkinetic model were found using a multi-dimensional Newton's method algorithm. The numerical 3-D volcano curve are obtained using the scaling relations[11, 12] between the adsorption energies of C and O and the adsorption energies of all the other species and energies of transition states. These methods are all implemented in the CatMAP[4-7] code, which is a python package developed in Nørskov's group for automatic generation of micro-kinetic models using the descriptor-based approach. The initial coverages for all the species were generated by CatMAP using Gibbs distribution, and 100 digits of precision were used to overcome the stiffness of the kinetic equations with the help of Python mpmath library[13]. More methods details in equation solving, linear regression, etc. can be found in the ref. [7].

Figure S1 The comparison of oxygen adsorption energies on the step edge of bi-phase surface, AB, ($E_O$bi-phase) and on the step edge of the mono A stepped surface ($E_O$mono). The point that falls far from the line was labelled.

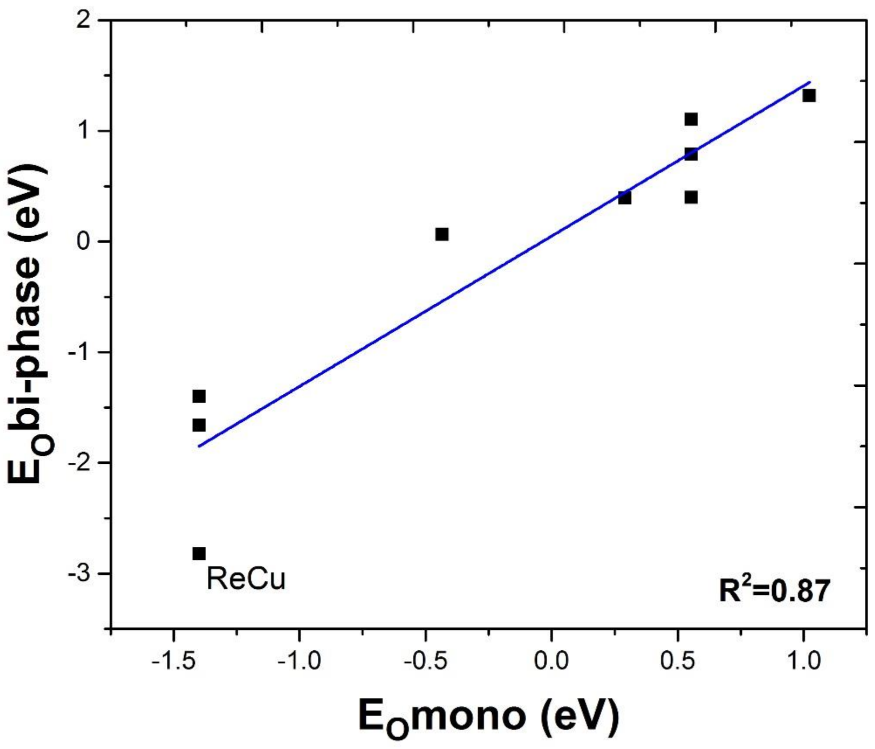

Table S1. The degrees of rate control of all the elementary steps in traditional microkinetic model on all the bi-phase surfaces.

	C* + O*	C* + OH*	C* + H*	CH* + H*	CH2* + H*	CH3* + H*	O* + H*	OH* + H*	O* + H2O*
RuRe	7.56E-02	1.04E-01	1.78E-03	2.30E-03	2.27E-03	4.97E-01	3.08E-01	1.10E-02	1.80E-02
RhRu	8.78E-01	1.24E-01	1.35E-03	1.71E-03	2.10E-03	2.28E-03	2.34E-03	2.72E-03	2.72E-03
ReRu	4.79E-03	3.77E-03	5.20E-03	7.54E-03	7.54E-03	7.54E-03	3.28E-01	7.55E-03	6.83E-01
RePt	9.19E-02	1.85E-05	2.20E-05	3.90E-05	4.87E-05	4.87E-05	9.07E-01	4.89E-05	9.18E-04
ReCu	5.05E-03	4.27E-03	6.37E-03	1.11E-02	1.11E-02	1.11E-02	1.01E+00	8.10E-03	4.34E-03
PtRe	1.89E-03	1.00E+00	1.03E-03	2.09E-03	1.70E-03	2.77E-03	1.39E-03	2.77E-03	1.72E-03
PtCu	8.60E-03	1.00E+00	1.15E-02	1.54E-02	2.11E-02	2.14E-02	3.01E-02	3.20E-02	2.73E-02
CuRe	-2.06E-03	2.06E-01	-3.07E-03	-3.85E-03	2.67E-01	-4.33E-03	-4.95E-03	5.65E-02	-4.66E-03
CuPt	-1.11E-03	-1.31E-03	9.24E-02	-7.88E-04	1.41E-01	-2.05E-03	-2.67E-03	7.52E-01	1.78E-03

Table S2 The reaction rates of overall products (overall), CH_4 (CH_4) and H_2O (H_2O) on different phase (Upper and Lower) on all the bi-phase surfaces. All the reaction rates are in s^{-1}.

	CH_4		H_2O		Overall
	Upper	Lower	Upper	Lower	Total
CuPt	1.66E-05	5.53E+00	5.53E+00	5.64E-12	5.53E+00
CuRe	6.47E-02	1.56E+00	1.63E+00	-2.57E-08	1.63E+00
PtCu	8.50E-18	3.02E-19	7.64E-29	8.80E-18	8.80E-18
PtRe	1.14E-05	1.09E-08	1.17E-07	1.13E-05	1.14E-05
ReCu	1.01E-24	8.80E-18	1.30E-28	8.80E-18	8.80E-18
RePt	1.64E-03	1.76E-04	1.82E-03	7.69E-16	1.82E-03
ReRu	1.55E-04	3.61E-02	5.29E-07	3.63E-02	3.63E-02
RhRu	2.33E-02	1.31E-02	6.92E-04	3.57E-02	3.64E-02
RuRe	5.30E+00	1.61E+00	6.90E+00	1.13E-05	6.90E+00

Table S3 Formation energies of all the intermediates and transition states on mono-phase surfaces. All the formation energies are with respect to the energies of H in H_2, C in CH_4, and O in H_2O, respectively.

	Cu	Pd	Pt	Re	Rh	Ru
C	3.14	1.35	2.00	0.78	1.21	1.52
CH	2.12	1.29	1.45	0.29	0.77	0.83
CH_2	1.73	1.08	0.54	0.21	0.81	0.65
CH_3	0.89	0.65	0.24	-0.32	0.47	0.35
CO	2.14	1.19	1.06	1.04	1.04	1.14
H	-0.24	-0.51	-0.64	-0.81	-0.58	-0.66
O	0.55	1.35	1.02	-1.40	0.29	-0.43
OH	-0.42	0.16	0.04	-1.32	-0.41	-0.87
C-H	3.84	1.79	2.50	1.07	1.57	1.77
CH-H	2.84	1.87	1.72	0.63	1.15	1.09
CH_2-H	1.90	1.19	0.48	0.01	0.68	0.52
CH_3-H	1.24	0.67	0.39	0.23	0.51	0.45
O-H	1.96	2.03	1.51	-0.35	1.04	0.33
OH-H	0.83	0.68	0.28	-0.26	0.34	0.10
O-H_2O	0.75	1.48	0.78	-1.90	0.44	-0.44
C-O	5.76	3.95	3.86	1.17	2.85	2.27
C-OH	4.52	3.03	3.18	1.54	2.49	2.15

Table S4 Formation energies of all the intermediates and transition states on bi-phase surfaces, including the upper, interface and lower reactions. All the formation energies are with respect to the energies of H in H_2, C in CH_4, and O in H_2O, respectively.

		CuPt	CuRe	PtCu	PtRe	ReCu	RePt	ReRu	RhRu	RuRe
C	upper	2.38	1.42	2.37	1.92	1.13	1.95	1.15	1.51	1.28
CH	upper	1.83	0.62	1.49	1.18	-0.61	1.23	0.42	0.94	0.65
CH_2	upper	1.60	1.54	0.66	1.25	-1.25	-0.28	-0.13	0.95	1.02
CH_3	upper	0.86	0.58	0.42	0.52	-1.16	-0.33	-0.05	0.54	0.30
CO	upper	2.08	2.13	1.08	1.49	-0.57	0.83	0.88	1.14	1.51
H	upper	-0.36	-0.45	-0.70	-0.30	-2.41	-1.05	-1.01	-0.57	-0.46
O	upper	0.79	0.40	1.10	1.32	-2.82	-1.40	-1.66	0.39	0.07
OH	upper	-0.36	-0.48	0.20	0.07	-2.88	-1.64	-1.71	-0.36	-0.47
C-H	upper	2.88	1.93	2.74	2.57	1.61	2.10	1.32	1.88	1.64
CH-H	upper	2.12	1.36	1.78	0.96	0.56	0.88	0.45	1.22	0.95
CH_2-H	upper	1.93	2.00	1.11	1.17	-0.27	-0.52	-0.30	0.84	0.95
CH_3-H	upper	1.13	0.85	0.86	0.22	-0.35	-0.14	0.20	0.51	0.78
O-H	upper	1.69	1.26	1.76	1.93	-0.66	-0.64	-0.65	1.10	0.78
OH-H	upper	0.91	0.46	0.80	0.06	-1.60	-0.64	-0.45	0.38	0.35
O-H_2O	upper	1.20	0.42	1.68	1.28	-2.74	-2.50	-2.26	0.38	-0.04
C-O	upper	3.94	2.94	6.09	3.51	1.90	2.11	1.28	2.81	2.10
C-OH	upper	2.66	2.02	5.33	2.93	2.35	2.38	1.75	2.52	1.90
H	interface	-0.43	-0.66	-0.04	-0.56	-1.43	-0.34	-0.39	-0.43	-1.39
O-H	interface	1.29	0.72	1.31	1.36	-1.35	-1.08	-0.99	0.50	0.11
OH-H	interface	0.15	-0.24	1.24	0.18	-1.05	-0.80	-0.40	0.32	-0.69
C	lower	2.00	0.78	3.14	0.78	3.14	2.00	1.52	1.52	0.78
CH	lower	1.45	0.29	2.12	0.29	2.12	1.45	0.83	0.83	0.29
CH_2	lower	0.54	0.21	1.73	0.21	1.73	0.54	0.65	0.65	0.21
CH_3	lower	0.24	-0.32	0.89	-0.32	0.89	0.24	0.35	0.35	-0.32
CO	lower	1.06	1.04	2.14	1.04	2.14	1.06	1.14	1.14	1.04
H	lower	-0.64	-0.81	-0.24	-0.81	-0.24	-0.64	-0.66	-0.66	-0.81
O	lower	1.02	-1.40	0.55	-1.40	0.55	1.02	-0.43	-0.43	-1.40
OH	lower	0.04	-1.32	-0.42	-1.32	-0.42	0.04	-0.87	-0.87	-1.32
C-H	lower	2.50	1.07	3.84	1.07	3.84	2.50	1.77	1.77	1.07
CH-H	lower	1.72	0.63	2.84	0.63	2.84	1.72	1.09	1.09	0.63
CH_2-H	lower	0.48	0.01	1.90	0.01	1.90	0.48	0.52	0.52	0.01
CH_3-H	lower	0.39	0.23	1.24	0.23	1.24	0.39	0.45	0.45	0.23
O-H	lower	1.51	-0.35	1.96	-0.35	1.96	1.51	0.33	0.33	-0.35
OH-H	lower	0.28	-0.26	0.83	-0.26	0.83	0.28	0.10	0.10	-0.26
O-H_2O	lower	0.78	-1.90	0.75	-1.90	0.75	0.78	-0.44	-0.44	-1.90
C-O	lower	3.86	1.17	5.76	1.17	5.76	3.86	2.27	2.27	1.17
C-OH	lower	3.18	1.54	4.52	1.54	4.52	3.18	2.15	2.15	1.54

References:

1. X. M. Cao, R. Burch, C. Hardacre and P. Hu, *Catal. Today*, 2011, **165**, 71-79.
2. X. M. Cao, R. Burch, C. Hardacre and P. Hu, *J. Phys. Chem. C*, 2011, **115**, 19819-19827.
3. T. Komatsu and A. Tamura, *J. Catal.*, 2008, **258**, 306-314.
4. C. M. Wang, R. Y. Brogaard, B. M. Weckhuysen, J. K. Norskov and F. Studt, *J Phys Chem Lett*, 2014, **5**, 1516-1521.
5. R. Y. Brogaard, R. Henry, Y. Schuurman, A. J. Medford, P. G. Moses, P. Beato, S. Svelle, J. K. Norskov and U. Olsbye, *J. Catal.*, 2014, **314**, 159-169.
6. A. J. Medford, J. Sehested, J. Rossmeisl, I. Chorkendorff, F. Studt, J. K. Norskov and P. G. Moses, *J. Catal.*, 2014, **309**, 397-407.
7. A. Medford, C. Shi, M. Hoffmann, A. Lausche, S. Fitzgibbon, T. Bligaard and J. Nørskov, *Catal. Lett.*, 2015, **145**, 794-807.
8. A. C. Lausche, A. J. Medford, T. S. Khan, Y. Xu, T. Bligaard, F. Abild-Pedersen, J. K. Nørskov and F. Studt, *J. Catal.*, 2013, **307**, 275-282.
9. K. Honkala, A. Hellman, I. N. Remediakis, A. Logadottir, A. Carlsson, S. Dahl, C. H. Christensen and J. K. Norskov, *Science*, 2005, **307**, 555-558.
10. Z. P. Liu and P. Hu, *J. Am. Chem. Soc.*, 2003, **125**, 1958-1967.
11. P. Ferrin, A. U. Nilekar, J. Greeley, M. Mavrikakis and J. Rossmeisl, *Surf. Sci.*, 2008, **602**, 3424-3431.
12. F. Abild-Pedersen, J. Greeley, F. Studt, J. Rossmeisl, T. R. Munter, P. G. Moses, E. Skulason, T. Bligaard and J. K. Norskov, *Phys. Rev. Lett.*, 2007, **99**, 016105.
13. J. F, *mpmath: a Python library for arbitrary-precision floating-point arithmetic (version 0.14)*, 2010.
